# Neural stem cell transplantation in progressive multiple sclerosis: STEMS study long-term outcomes

**DOI:** 10.1186/s13287-026-05148-5

**Published:** 2026-07-22

**Authors:** Maria A. Rocca, Angela Genchi, Matteo Azzimonti, Irene Gattuso, Elisabetta Pagani, Loredana Storelli, Paolo Vezzulli, Elena Brambilla, Roberto Santangelo, Raffaella Greco, Fabio Ciceri, Roberto Furlan, Lucia Moiola, Andrea Falini, Gianvito Martino, Massimo Filippi

**Affiliations:** 1 Neuroimaging Research Unit, Division of Neuroscience, IRCCS San Raffaele Scientific Institute, Milan, Italy; 2 Neurology Unit, IRCCS San Raffaele Scientific Institute, Via Olgettina, 60, 20132 Milan, Italy; 3 Vita-Salute San Raffaele University, Milan, Italy; 4 Neuroimmunology Unit, Institute of Experimental Neurology, Division of Neuroscience, IRCCS San Raffaele Scientific Institute, Milan, Italy; 5 Neuroradiology Unit, IRCCS San Raffaele Scientific Institute, Milan, Italy; 6 Neurophysiology Service, IRCCS San Raffaele Scientific Institute, Milan, Italy; 7 Haematology and Bone Marrow Transplantation Unit, IRCCS San Raffaele Scientific Institute, Milan, Italy; 8 Clinical Neuroimmunology Unit, Institute of Experimental Neurology, Division of Neuroscience, IRCCS San Raffaele Scientific Institute, Milan, Italy; 9 Neurorehabilitation Unit, IRCCS San Raffaele Scientific Institute, Milan, Italy

**Keywords:** Multiple sclerosis, Stem cells, Transplantation, Biomarkers, Brain atrophy

## Abstract

**Background:**

To evaluate the long-term safety and potential neuroprotective effects of human fetal neural precursor cell transplantation in individuals with progressive multiple sclerosis.

**Methods:**

This retrospective observational study assessed long-term follow-up data (median 5.7 years) from participants in a first-in-human phase I trial (NCT03269071) investigating neural cell transplantation in progressive multiple sclerosis. Clinical and radiological available data were collected and compared to baseline values and reference populations.

**Results:**

Long-term follow-up data were available for 11 of 12 patients. No serious adverse events related to cell transplantation occurred, confirming long-term safety. Although physical disability progressed over time, cognition remained stable compared to baseline. Five patients developed new T2-hyperintense brain lesions without associated contrast enhancement. Long-term gray matter atrophy rates were lower than those observed during the initial two years post-transplantation. Rates of gray and white matter atrophy approximated those of age-matched healthy individuals and patients with early-stage multiple sclerosis, with a trend for a lower atrophy rate compared to a control cohort with progressive disease. These structural findings were consistent with stable levels of circulating biomarkers of neurodegeneration.

**Conclusions:**

The findings support the long-term safety of fetal neural cell transplantation in progressive multiple sclerosis. Although exploratory in nature, the stability of cognitive function, reduced brain atrophy, and consistent biomarker profiles support the hypothesis of a possible neuroprotective effect that warrants further investigation in a controlled clinical trial setting.

**Supplementary Information:**

The online version contains supplementary material available at https://doi.org/10.1186/s13287-026-05148-5.

## Background

Development of effective therapies for people with progressive multiple sclerosis (PMS) still represents an unmet need. PMS is characterized by neurodegenerative phenomena closely related to the exhaustion of plasticity mechanisms and endogenous repair processes, leading to irreversible disability accrual [1]. The neuroprotective and regenerative potential of neural precursor cells (NPCs) has been widely demonstrated in preclinical studies, paving the way for clinical trials in MS [2–6]. In MS models, transplanted NPCs migrate into damaged central nervous system (CNS) areas, promoting regeneration and immune modulation with clinical and neurophysiological benefits [5, 6]. These positive effects are mainly driven by the bystander effect, with NPCs remaining undifferentiated and releasing a milieu of immunomodulatory and neurotrophic factors promoting endogenous repair [6], rather than by direct cell replacement [7].

The first-in-human STEMS phase I study demonstrated the safety of human fetal NPCs (hfNPCs) intrathecal transplantation in PMS and provided preliminary evidence of neuroprotection. Exploratory magnetic resonance imaging (MRI) evidence of a significant lower rate of brain atrophy in patients receiving the highest dose of hfNPCs, supported by immunological and proteomic cerebrospinal fluid (CSF) shift towards a more pro-regenerative profile, support an hfNPC-mediated bystander neuroprotective effect [8]. These results were later confirmed in a phase I study evaluating intra-cerebroventricular hfNPCs injection [9]. These findings provide the rationale for a phase II clinical trial evaluating the efficacy of hfNPCs in a larger PMS cohort. Since the development of academic, no-profit clinical studies on advanced cellular therapies requires considerable time [10], reassessing the long-term phase I outcomes in parallel with the phase II development is crucial. We followed transplanted patients over time to detect late adverse events (AEs) and to assess a possible long-lasting neuroprotective effect. This retrospective observational study provides long-term clinical, neurophysiological and MRI evaluation of patients enrolled in STEMS phase I study.

## Methods

### STEMS phase I trial design and population

STEMS was a prospective, non-randomized, single-centre, open-label phase I clinical trial (NCT03269071, EudraCT2016-002020-86) evaluating safety of hfNPC transplantation in PMS [8]. Patients were enrolled according to inclusion and exclusion criteria, as follows: (1) age 18–55 years; (2) diagnosis of MS according to revised 2010 McDonald criteria [11], with a progressive course according to 2013 Lublin definition [1]; (3) failure or intolerance to all approved therapies; (4) Expanded Disability Status Scale (EDSS) score [12] ≥ 6.5 and disease duration 2–20 years; (5) evidence of progression of the disease, defined by an increase of ≥ 0.5 EDSS points in the last 12 months; (6) presence of oligoclonal bands in the CSF required for primary PMS; (7) no treatment with any immunosuppressive therapy within 3 months before screening or with interferon-beta, glatiramer acetate or corticosteroids within 30 days before screening; (8) no relapses within 30 days before screening.

Briefly, STEMS-patients were consecutively enrolled in 4 treatment cohorts (TCs). Given the first-in-human, phase I nature of the study, a standard 3 + 3 design, was used. Each TC received a single intrathecal administration of one of the four increasing treatment doses of hfNPCs (TC-A: 0.7 × 10^6^, TC-B: 1.4 × 10^6^, TC-C: 2.8 × 10^6^ and TC-D: 5.7 × 10^6^ ± 10% viable cells per kilogram of body weight), derived from a single 10–12 weeks post-conception human fetus (elective abortion), expanded under good manufacturing practice conditions, and administered through lumbar puncture. To prevent rejection of transplanted hfNPCs, they received immune suppression with tacrolimus from day 7 before transplantation until week 96 after. All patients in the trial discontinued tacrolimus at the end of the 2-year follow-up, as per protocol, or according to the timelines reported in the phase I trial for specific cases. Primary outcome was to assess feasibility, safety and tolerability of the procedure over a period of 96 weeks. Secondary exploratory outcomes included longitudinal variations from baseline (pre-hfNPC administration) of clinical (EDSS), neuropsychological (Symbol Digit Modalities Test [SDMT]), optical coherence tomography (OCT), plasma biomarkers and MRI variables over a period of 96 weeks of follow-up (2Y-FU). The results of this phase I clinical trial have already been described [8].

### Long term follow-up design and population

This retrospective observational study collected long-term follow-up (LT-FU) data of the patients enrolled in the phase I study, who underwent continuous monitoring according to clinical practice. All clinical assessments (including neurological examinations, SDMT, 9HPT, and adverse events) were collected retrospectively from the medical records, as documented during routine clinical visits. STEMS-patients with an available neurological visit and MRI performed according to clinical practice at least 4.5 years after hfNPC transplantation were assessed. OCT scans were evaluated when available. Available serum samples collected and stored in the INSPE Biobank during routine clinical care and obtained at least 4.5 years after the hfNPC transplantation have been analyzed for neurofilament light chain sNfL, tau, glial fibrillary acidic protein (GFAP), ubiquitin C-terminal hydrolase-L1 (UCH-L1) content. Retrospective data have been collected from the end of the phase I study to July 2024. Although a baseline MRI scan prior to hfNPC transplantation was available for all STEMS patients, a standardized pre-transplant MRI assessment was lacking, precluding direct estimation of individual atrophy rates before transplantation. For this reason, demographic, clinical and MRI data from three longitudinal cohorts – healthy controls (HC), early MS, and PMS - were retrospectively collected as a reference for atrophy rates at LT-FU.

### Clinical evaluation

At LT-FU visit (last available neurological visit according clinical practice at least 4.5 years after transplantation), STEMS-patients underwent a comprehensive neurological evaluation with collection of demographics (age and sex) and clinical variables, including clinical relapses, possible disease modifying treatment initiation and EDSS score [12]. AEs were collected and their rate and nature have been classified according to the Common Terminology Criteria for Adverse Events version 5.0 and attributed to the investigational medicinal product accordingly to National Cancer Institute guidelines of AE reporting requirements. As a measure of cognitive impairment, the oral SDMT has been collected to quantify the speed of information processing, by asking the patient to associate symbols and numbers as quickly as possible, an essential cognitive function early affected in MS [13, 14]. The patient’s score is estimated as the number of correct associations produced in 90 s. The 9-HPT evaluates hand function and coordination. To conduct this timed test, the patients are seated with a peg board in front of them. The board has nine holes on one side and a shallow round dish on the other side, and the patients are asked to place the pegs into each hole on the peg board, one at a time, and remove them again one at a time, while the test speed is measured. The patients were tested twice for each arm, the dominant arm first. An average time was calculated separately for the dominant and non-dominant hands.

### OCT

OCT was performed using spectral domain Spectralis OCT (Heidelberg Engineering, software version 5.8). Standard peripapillary retinal nerve fiber layer (pRNFL) was measured on a standard 3.5-Ø-mm circle scan protocol centered on the optic disc by the scanning technician. Global pRNFL thickness was automatically measured through the pre-installed software provided by the manufacturer. As previously described [8], for subjects with a history of unilateral optic neuritis (ON), we studied pRNFL of unaffected eye, while for subjects with an history of bilateral or no ON, we considered pRNFL mean of both eyes.

### Reference cohorts for the evaluation of atrophy

Since standardized MRI scans of STEMS-patients before the transplantation were lacking, we retrospectively collected demographic, clinical and MRI data from three longitudinal cohorts as a reference for atrophy rates at LT-FU (Table 3). The MRI data related to the year prior to NPCs transplantation summarized in Fig. 2a were collected retrospectively and refer exclusively to the presence of new T2 lesions or gadolinium-enhancing lesions. Since these MRIs were performed by using scanners and acquisition protocols different from those used in the study, they do not allow for a longitudinal analysis of brain volumes. From the local database at the Neuroimaging Research Unit of IRCCS San Raffaele Scientific Institute in Milan, we selected:

(1) 55 patients studied at time of their clinically isolated syndrome suggestive of MS (early MS). Inclusion and exclusion criteria were: age between 18 and 60 years, a typical clinical presentation of relapsing-onset MS, baseline neurological evaluation and brain MRI performed within 5 months of the clinical onset. Patients underwent clinical and brain MRI evaluation after 2-years of follow-up; (2) 19 age- and sex-matched PMS patients (3 PPMS and 16 SPMS) with a neurological evaluation and brain MRI performed both at baseline and after at least one year of follow-up. The 58% of PMS patients were under treatment with disease modifying therapies; (3) 23 age-matched HC with the following inclusion criteria: (a) age ≥ 45 years at baseline; (b) at least 2 longitudinal MRI acquisitions on the same scanner, with a minimum follow-up of 1.5 years; (c) no history of major neurological or psychiatric disorder.

### MRI acquisition and analysis

All brain MRI scans of STEMS-patients and early MS were acquired on a 1.5-Tesla MR scanner (Achieva dStream, Philips Medical Systems), with the following sequences, respectively for the two cohorts: 3D or 2D fluid-attenuated inversion recovery (FLAIR): repetition time (TR) = 4800 or 8000 ms, echo time (TE) = 277 or 90 ms, inversion time (TI) = 1660 or 2500 ms, echo train length (ETL) = 171 or 9, flip angle (FA) = 90°, field of view (FOV) = 230 × 230 × 184 mm^3^ or 250 × 250 mm^2^, 160 sagittal or 44 axial slices, voxel size = 1.15 × 1.15 × 1.15 mm^3^ or 0.98 × 0.98 mm^2^ with slice thickness = 3 mm and gap = 1 mm, matrix = 200 × 200 × 160 or 256 × 256, number of signal averages (NSA) = 2 or 1; 2D T2-weighted turbo spin echo (TSE): TR = 4446 or 3900 ms, TE = 100 or 90 ms, ETL = 15 or 6, FA = 90°, FOV = 230 × 192 mm^2^ or 250 × 250 mm^2^, 44 axial slices; slice thickness = 5–3 mm, gap = 1 mm, pixel size = 0.6 × 0.9 mm^2^ or 0.98 × 0.98 mm^2^, matrix = 384 × 224 or 256 × 256, NSA = 3 or 1; 3D T1-weighted turbo field echo (TFE) or magnetization-prepared rapid gradient echo (MPRAGE): TR = 8 ms, TE = 3.7 ms, TI = 1000 ms, ETL = 171, FA = 8°, FOV = 256 × 256 × 192 mm^3^, 160 sagittal slices, voxel size = 1.0 × 1.0 × 1.2 mm^3^, matrix = 256 × 256 × 160, NSA = 1; Post-contrast 2D T1-weighted TSE: TR = 580 or 600 ms, TE = 12 or 15 ms, FA = 69°, FOV = 230 × 183 mm^2^ or 250 × 250 mm^2^, 22 or 44 axial slices, slice thickness = 5–3 mm, gap = 1 mm, pixel size = 0.85 × 1.12 mm^2^ or 0.98 × 0.98 mm^2^; matrix = 272 × 164 or 256 × 256, NSA = 2, 5 min after the injection of gadobutrol 0.1 ml/kg (for STEMS-patients, see [8] for further details on the MRI protocol). STEMS patients underwent MRI acquisition at baseline (pre-hfNPC administration) and at 96 weeks of follow-up (2Y-FU). For LT-FU, the last available MRI scan acquired according to clinical practice on the same MR scanner (Achieva dStream, Philips Medical Systems) and performed at least 4.5 years after hfNPC transplantation was assessed.

The brain MRI scans of HC and PMS were acquired on a Intera and Achieva (Philips Medical Systems, Best, The Netherlands) or a Signa HDxt (GE Healthcare, Milwaukee, USA) 3.0-Tesla scanners. 3D T1-weighted and T2-weighted scans were acquired using the following protocol (both at baseline and at follow-up): 3D T1-weighted TFE or inversion recovery fast spoiled gradient echo (IR–FSPGR): TR = 7 ms, TE = 2.85 to 3.2 ms, TI = 650 to 900 ms, FA = 8°, FOV = 256 × 240 or 256 × 256, voxel size = 1.0 × 1.0 × 1.0 mm^3^ or 1.0 × 1.0 × 1.2 mm^3^; 2D dual-echo or FLAIR T2-weighted sequence: TR = ranging from 2900 to 9002 ms, TE = 16 to 120 ms, FA = 90°, FOV = 243 × 243 mm^2^ or 256 × 256 mm^2^; slice thickness = 3 mm, pixel size = 1 × 1 mm^2^.

At follow-up, subjects were carefully repositioned, with slices running parallel to a line that joins the most infero-anterior and infero-posterior parts of the corpus callosum, covering the entire brain from the skull base to the vertex.

For MRI analyses, all sagittal acquisitions were reoriented in axial plane and the exceeding portion of the neck was removed.

*Focal lesion assessment*. In STEMS-, early MS and PMS patients, focal brain T2-hyperintense white matter (WM) lesions were identified using a fully automated deep-learning approach on co-registered 3D FLAIR and 3D T1-weighted input images (nicMSlesions, relase 0.2, Python version 2.7) [15]. Lesion segmentation masks were carefully reviewed by an experienced observer, blinded to patients’ identity, and eventually edited to remove false-positive lesions. For HC, the presence of WM lesions was visually checked and eventually segmented by the same experienced observer. T2-hyperintense lesion volume was then obtained from lesion segmentation masks. For STEMS-, early MS and PMS patients, the number of new/enlarging brain T2-hyperintense lesions were counted in respect to the previous visit. The percentage of T2-hyperintense lesion volume change was also obtained. At all timepoints, Gd-enhancing lesions were manually counted on post-contrast T1-weighted scans.

### Brain atrophy analysis

Whole-brain, gray matter (GM) and WM normalized volumes were measured on 3D T1-weighted images using the SIENAX version 2.6, FSL 5.0.1 software [16], after applying lesion filling technique to replace intensity values within lesions so that they resemble normal tissue [17]. The percentage brain volume changes (PBVCs) were estimated at follow-up timepoint vs. the previous visit using the FSL SIENA software [16]. The percentage of GM and WM volume changes (PGMVC and PWMVC) were calculated using the segmentation tool and pairwise longitudinal registration in the Statistical Parametric Mapping (SPM) toolbox version12, MATLAB version 8.0, to segment the tissues and quantify volume changes from images of the Jacobian determinants derived from the non-linear registration between two visits [18, 19]. All percentage of longitudinal brain tissues volume changes were annualized, dividing by the follow-up duration.

For a better visual assessment of brain atrophy rates over time after transplantation, MRI PBVC, PGMVC and PWMVC quantifications at week 48 of the STEMS protocol were also retrieved.

### Plasma biomarker analysis

Available samples collected and stored in the INSPE Biobank at least 4.5 years after the hfNPC transplantation have been analyzed for sNfL, tau, GFAP, UCH-L1 content using Simoa Human Neurology 4-Plex A (N4PA, 102153) digital immunoassay on an HD-1 single-molecule array (Simoa) instrument according to instructions from the manufacturer (Quanterix). The measurements were performed by a technician blinded to clinical data, in one round of experiment using one batch of reagents. Plasma samples were diluted four times. This is a two-steps immunoassay, in which target antibody coated paramagnetic beads are combined with sample and biotinylated detector antibody in the same incubation. Target molecules present in the sample are captured by the antibody-coated beads and bind with the biotinylated antibody detector simultaneously. After a wash, a conjugate of streptavidin-ß-galactosidase (SBG) is mixed with the beads. SBG binds to the biotinylated detector antibodies, resulting in enzyme labelling of the captured target. After a final wash, the beads are resuspended in a resorufin ß-d-galactopyranoside (RGP) substrate solution and transferred to the Simoa Disc. Individual beads are then sealed within microwells in the array. If the target has been captured and labeled on the bead, ß-galactosidase hydrolyzes the RGP substrate in the microwell into a fluorescent product that provides the signal for measurement. A single-labeled target molecule results in sufficient fluorescent signal in 30 s to be detected and counted by the Simoa optical system.

### Statistical analysis

Statistical analyses and graphs were produced using R software (version 4.3.3) and with GraphPad Prism (version 8.0, GraphPad Software). All variables are summarized as median values or changes (with inter-quartile ranges) or absolute numbers (and percentages), according to the type of data.

Clinical, biological and neurophysiological variables of STEMS-patients were compared over time using a Wilcoxon Signed Ranks Test for paired samples. The yearly rate of changes (slope) after transplantation of EDSS, SDMT and pRNFL measures were estimated within subjects. The subjects’ specific slopes two years after transplantation and between LT-FU and 2Y-FU were compared by a non-parametric exact Wilcoxon test.

Demographic variables were compared among STEMS-patients and reference cohorts using Fisher’s exact test for categorical variables and the Independent-Samples Kruskal-Wallis test with post-hoc comparisons for continuous variables. Changes of all MRI measures were estimated between LT-FU and 2Y-FU for STEMS-patients, and between follow-up and baseline visit for reference cohorts. The within-patient longitudinal changes of MRI variables were compared by a non-parametric exact Wilcoxon Signed Ranks Test for paired samples. The associations of longitudinal variations of clinical and MRI measures with the number of injected cells at baseline were obtained using multivariate regression analysis, adjusting for baseline scores. Longitudinal changes of clinical and MRI variables among STEMS-patients and reference cohorts were compared using multivariate ANCOVA models corrected for age, sex, normalized brain tissue volumes, EDSS and MR scanner (as appropriate). To account for the potential imbalance in clinical phenotype distribution between STEMS and PMS groups, a sensitivity analysis was performed including clinical phenotype (SPMS vs. PPMS) as an additional covariate in the statistical models.

Longitudinal comparisons of clinical and MRI variables between LT-FU and 2Y-FU were performed only on the STEMS-patients with available LT-FU data (11/12). P values less than 0.05 were considered statistically significant. Owing to the exploratory nature of the analyses, no corrections for multiple testing were performed, and P values must be considered as descriptive.

## Results

### Patient characteristics and safety outcome

Detailed characterization of the trial population is reported in Table 1.

**Table 1:** Clinical and demographic features of the STEMS-patients

	STEMS population			
	Baseline			
Female Sex - n (%)	8 (66.7)			
Age (years) - Mean ± SD	47.7 ± 4.9			
Disease type				
SPMS - n (%)	5 (41.7)			
PPMS - n (%)	7 (58.3)			
Disease Duration (years) - Mean ± SD	14.6 ± 4.9			
Previous DMT - n (%)				
AZA	2 (16.7)			
MTX + CTX	1 (8.3)			
FTY	4 (33.3)			
RTX/OCR	2 (16.7)			
CTX	1 (8.3)			
DMF	2 (16.7)			
Time from last DMT to transplantation (years) - Mean ± SD				
	2.9 ± 1.8			

Clinical and Neurophysiological assessment	Baseline (*n* = 12)	2Y FU (*n* = 12)	LT FU (*n* = 11)	p value
EDSS score (median [IQR])	7.25 [6.5-8.0]	7.75 [7.0–8.0]	8.5 [8.0–9.0] (*n* = 11)	LT vs. Baseline; LT vs. 2Y *p* < 0.01
SDMT score (median [IQR])	39.5 [34.3–51]	50 [43–51] (*n* = 11)	42 [32-46.3] (*n* = 10)	LT vs. Baseline *p* = 0.92; LT vs. 2Y *p* < 0.01
pRNFL thickness, µm (mean ± SD)	91.2 ± 11.9 (*n* = 12)	90.7 ± 13.3 (*n* = 11)	92.9 ± 9 (*n* = 8)	LT vs. Baseline *p* = 0.05; LT vs. 2Y *p* = 0.3

SPMS: secondary progressive multiple sclerosis; PPMS: primary progressive multiple sclerosis; DMT: disease modifying therapy; AZA: azathioprine; MTX: Methotrexate; FTY: Fingolimod; RTX: rituximab; OCR: ocrelizumab; CTX: cyclophosphamide; DMF: Dimethyl Fumarate; EDSS: Expanded Disability Status Scale; IQR: Interquartile Range; SDMT: symbol digit modalities test; pRNFL: Peripapillary retinal nerve fiber layer

Complete LT-FU data were available for 11/12 (91.7%) patients with a median follow-up duration of 5.7 years after the hfNPCs transplantation (interquartile range [IQR] 4.8-6.0 years). The twelfth patient discontinued follow-up visits at our center due to disability-related logistical constraints. No AEs related to the procedure were observed. The most relevant AEs observed are detailed in Table 2.

**Table 2:** Significant adverse events retrospectively collected at long-term follow-up of STEMS phase I study patients

AEs Description	Patient’s ID and TC	Severity	Action taken	Outcome	Causal relationship to study procedure
MGUS IgG kappa at low risk of progression	#001, TC-1	Grade1	FU	Ongoing	Unlikely
Hypertransaminasemia	#004, TC-1	Grade1	FU	Spontaneously resolved	Unlikely
Post-traumatic tibial fracture	#007, TC-2	Grade3*	Surgery	Resolved	Unlikely
Post-traumatic spinal and pelvic fracture	#010, TC-3	Grade3*	Surgery	Resolved	Unlikely
Breast ductal carcinoma in situ	#013, TC-4	Grade3	Surgery and RT	Complete remission	Unlikely
Angiomyolipoma enlargement	#015, TC-4	Grade3	TAE	Volumetric reduction	Unlikely

AEs: Adverse events; TC: treatment cohort (as reported in the online methods section, STEMS-patients were consecutively enrolled in 4 treatment cohorts (TCs), each receiving a single intrathecal administration of one of the four increasing treatment doses of hfNPCs (TC-A: 0.7 × 10^6^, TC-B: 1.4 × 10^6^, TC-C: 2.8 × 10^6^ and TC-D: 5.7 × 10^6^ ± 10% viable cells per kilogram of body weight); MGUS: Monoclonal gammopathy of undetermined significance; FU: Follow-up; RT: radiotherapy; TAE: transarterial Embolization

*Limiting self-care ADL

No MS relapses occurred during LT-FU. 4/11 (36%) patients started a disease-modifying treatment (DMT) after discontinuation of tacrolimus according to STEMS protocol; of these, 1 started teriflunomide due to minimal radiologic disease activity (a small brain lesion without gadolinium -gd- enhancement) during the LT-FU, and 3 started ocrelizumab. Reasons for starting ocrelizumab were clinico-radiological activity during STEMS phase I protocol (*n* = 1) or radiological activity during STEMS LT-FU (*n* = 2).

### Clinical, neuropsychological and neurophysiological outcomes

**Fig. 1:**
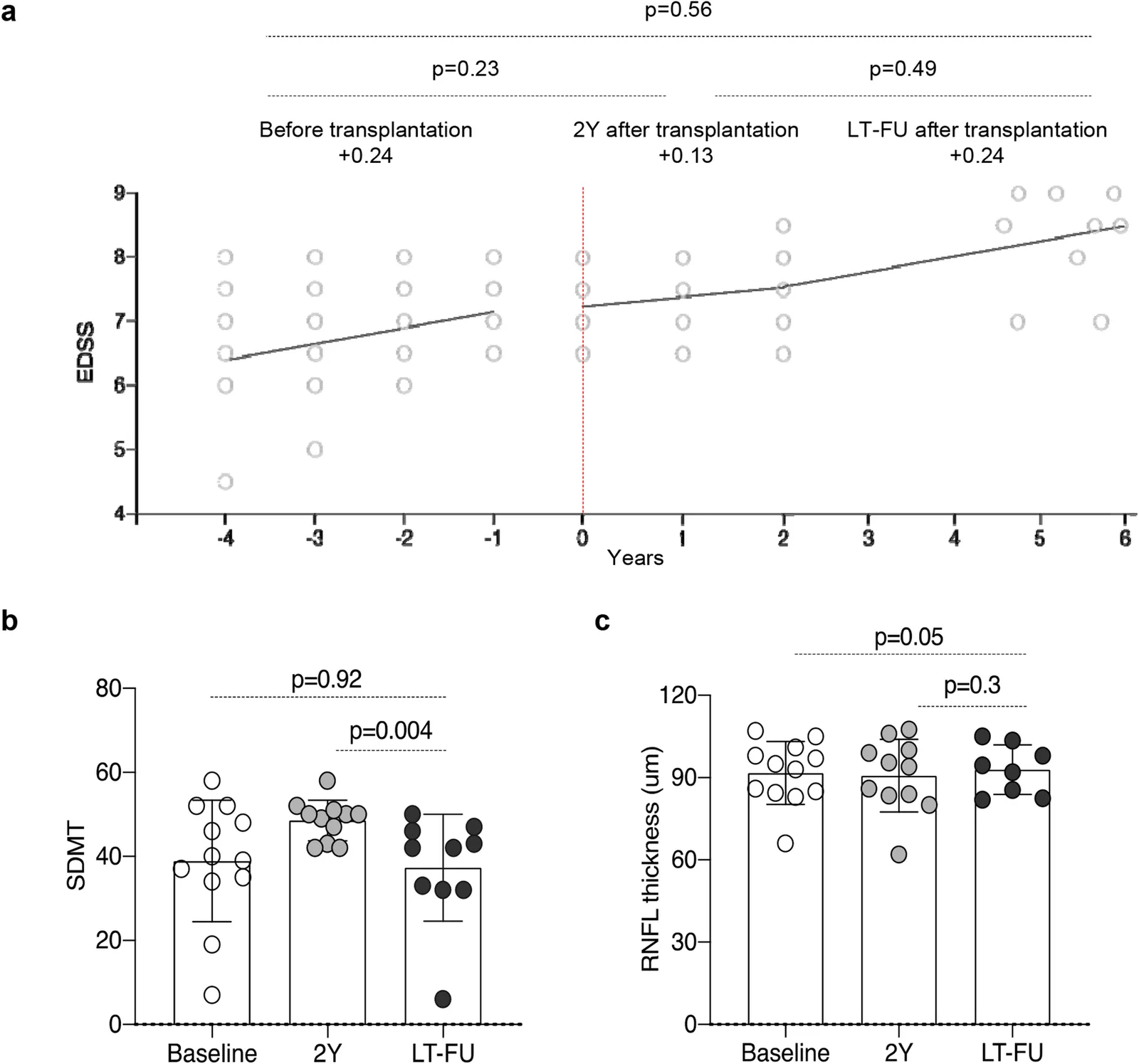
Clinical, cognitive and neurophysiological evolution of STEMS patients over time. **a** Slope of EDSS before transplantation, during the 2-year and LT-FU; the intra-patient annualized EDSS changes were compared by an Exact Wilcoxon test. Dot plots representing SDMT (**b)** and global medium RNFL thickness (**c**) at baseline, 2-year and LT-FU. EDSS: expanded disability status scale; Y: years; LT-FU: long-term follow-up; SDMT: symbol digit modalities test; RNFL: Retinal Nerve Fiber Layer; Yrs: years

EDSS in STEMS-patients significantly worsened at LT-FU (median EDSS [IQR]: 8.5 [8.0–9.0]) compared to baseline (median EDSS [IQR]: 7.25 [6.5-8.0]) and 2-year FU (median EDSS [IQR]: 7.75 [7.0–8.0]. *P* < 0.01, for both comparisons). The mean rate of EDSS change (slope) in the LT-FU (+ 0.24 EDSS points per year [range, 0–0.63]) did not significantly differ from the slope in the 4 years before (+ 0.24 EDSS points per year [range, 0–0.70]; *P* = 0.56) and 2 years after transplantation (+ 0.13 EDSS points per year [range, 0–0.80]; *P* = 0.49) (Fig. 1a). Out of 11 patients, only 4 and 3 could perform the nine-hole peg test with their dominant (DH) and non-dominant hand (NDH), respectively, suggesting an overall worsening in manual dexterity. Among these, 3 for DH and 1 for NDH experienced a score decline exceeding 20%. The LT-FU SDMT score (median SDMT 42, IQR 32.5–46.5) significantly worsened compared to 2-year FU (median SDMT 49, IQR 42.5–50; *P* < 0.01), whereas it remained stable compared to baseline visit (median SDMT 38, IQR 30.15-49; *P* = 0.92; Fig. 1b).

Long-term OCT assessment was available for 8/11 patients. pRNFL thickness values at LT-FU (mean ± SD pRNFL thickness 92.9 ± 9 μm) showed no significant differences compared to baseline (mean ± SD pRNFL thickness 91.7 ± 11.5 μm, *P* = 0.05) and 2Y-FU (mean ± SD pRNFL thickness 90.7 ± 13.3 μm, *P* = 0.30; Fig. 1c). No significant correlation was found between number of injected hfNPCs and slopes of EDSS (Spearman R^2^ = 0.19, *P* = 0.12), SDMT (Spearman R^2^ = 0.26, *P* = 0.09) and pRNFL (Spearman R^2^ = 0.19, *P* = 0.65) between LT-FU and 2Y-FU.

### MRI assessments and reference populations

**Fig. 2:**
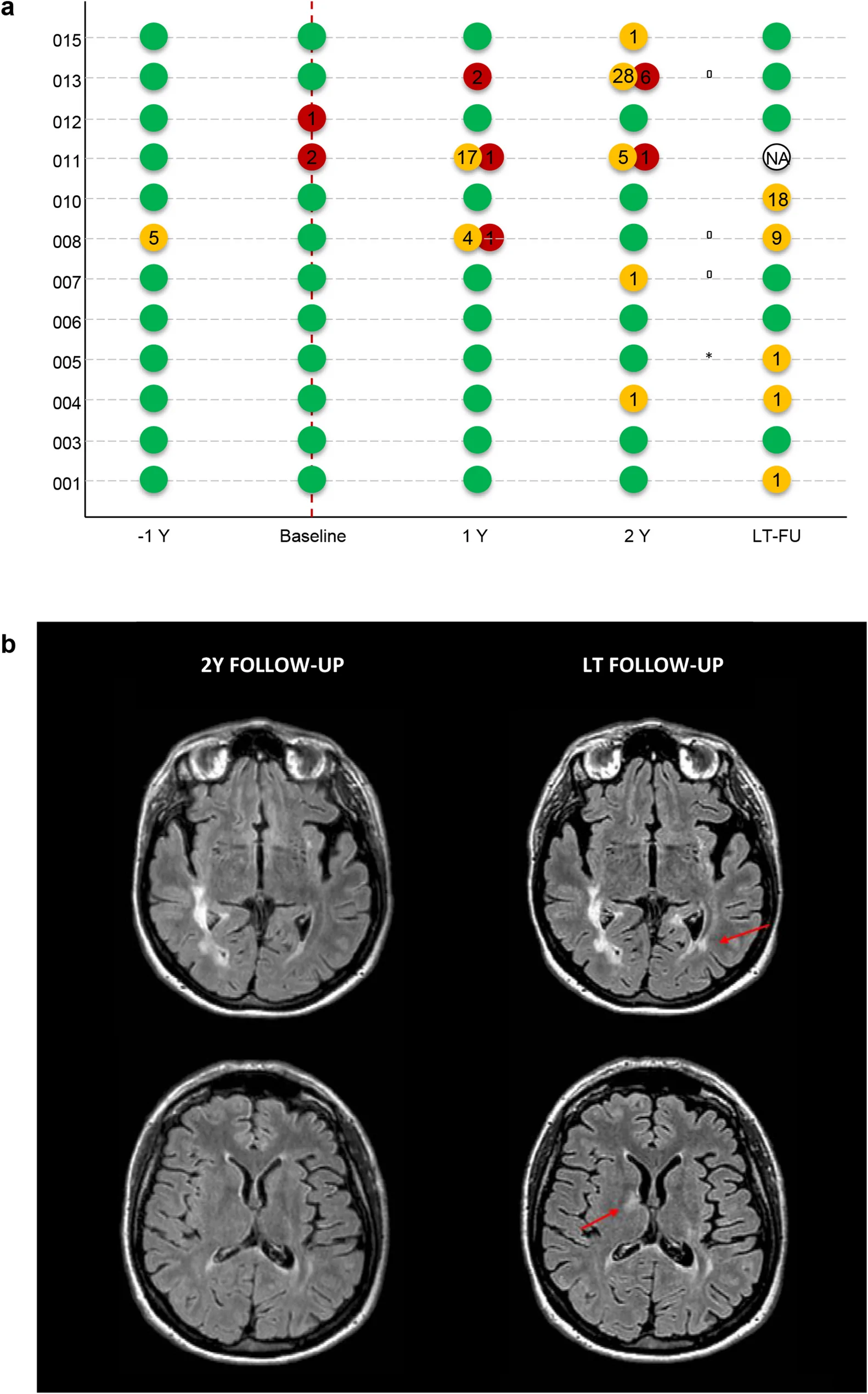
MRI inflammatory activity of STEMS patients over time. **a** Representation of patient-level MRI inflammatory activity over time. Red dots represent gadolinium enhancing lesions, orange dots new/enlarging T2-hyperintense lesions, green dots absence of MRI inflammatory activity. White dot, not available time point. The MRI data related to the year prior to NPCs transplantation were collected retrospectively and refer exclusively to the presence of new T2 lesions or gadolinium-enhancing lesions. **b** On the left side, representative axial FLAIR images from 2-years (2Y) follow-up of a single patient; on the right side, co-registered axial FLAIR images from long-term (LT) follow-up of the same subject showing the appearance of 3 new T2-hyperintense lesions (red arrow). Yrs: years; LT-FU: long-term follow-up; FLAIR: fluid attenuated inversion recovery; *initiation of teriflunomide; °initiation of anti-CD20

At LT-FU, 5 (45%) STEMS-patients showed new/enlarged T2-hyperintense brain lesions (Fig. 2, patient-by-patient representation of MRI inflammatory activity) with a median annualized percentage change in T2-hyperintense lesion volume (T2LV) of 8.94 (IQR − 1.56–14.88) %. Three of these 5 patients were not active at previous MRI timepoints. The proportion of STEMS-patients showing MRI disease activity and the annualized variation of total T2LV was not significantly different between LT-FU vs. 2Y-FU (*P* = 0.83 and *P* = 0.12, respectively; Table 3).

**Table 3:** Demographic and MRI variables of STEMS-patients and reference cohorts

Variables	STEMS 2Y-FU (*n* = 11)	STEMS LT-FU (*n* = 11)	Early MS (*n* = 55)	PMS (*n* = 19)	HC (*n* = 23)	2Y-FU *p*	LT-FU *p*
Median age (IQR) [years]	51.5 (45.5; 55.0)		32.1 (28.2; 39.5)	51.5 (36.1; 57.5)	50.7 (47.0; 53.5)	*vs. early MS*: <0.001^a^ *vs. PMS*: 0.57 ^a^ *vs. HC*: 0.51^a^	
Sex (%) [M/F]	4/7 (64/36)		26/29 (47/53)	6/13 (32/68)	11/12 (52/48)	*vs. early MS*: 0.50^b^ *vs. PMS*: 0.87 ^b^ *vs. HC*: 0.72^b^	
Median follow-up duration^#^ (IQR) [years]	1.9 (1.8; 2.0)	3.8 (2.8; 4.1)	2.0 (1.9; 2.1)	1.8 (1.1; 4.1)	2.2 (1.4; 6.0)	*vs. early MS*: 0.98^a^ *vs. PMS*: 0.51^a^ *vs. HC*: 0.06^a^	*vs. 2Y*: <0.01^c^ *vs. early MS*: <0.001^a^ *vs. PMS*: <0.001^a^ *vs. HC*: <0.01^a^
Median disease duration (IQR) [years]	14.0 (12.0; 19.0)		0.1 (0; 0.2)	14.0 (8.26; 19.5)	-	*vs. early MS*: <0.001^a^ *vs. PMS*: 0.73^a^	
Median EDSS (IQR)	7.75 (7.0–8.0)	8.5 (7.75; 9.0)	1.0 (0; 1.5)	6.0 (6.0; 6.5)	-	*vs. early MS*: <0.001^a^ *vs. PMS*: <0.01^a^	*vs. 2Y*: <0.01^c^ *vs. early MS*: <0.001^a^ *vs. PMS*: <0.001^a^
N. patients on DMT (%) [none/moderate efficacy/high efficacy^§^]	12/0/0	7/1/3 (64/9/27)	33/19/3 (60/35/5)	8/7/4 (42/37/21)	-	*-*	*-*
N of patients showing new/enlarged T2 brain lesions (%)	5 (46%)	5 (46%)	5 (9%)	9 (47%)	-	*vs. early MS*: <0.01^b^ *vs. PMS*:0.43^b^	*vs. 2Y*: 0.99^b^ *vs. early MS*: 0.01^b^ *vs. PMS*:0.43^b^
Median change in T2LV^*^ (IQR)	4.6 (-0.9; 32.3)	8.94 (-1.56; 14.88)	-2.0 (-6.2; 6.1)	0.1 (0.0; 0.1)	-	*vs. early MS*: <0.01^a^ *vs. PMS*: <0.001^b^	*vs. 2Y*: 0.12^c^ *vs. early MS*: <0.01^a^ *vs. PMS*: <0.001^b^
Median PBVC^*^ (IQR)	-0.73 (-1.41; -0.45)	-0.56 (-1.24; -0.44)	-0.19 (-0.67; -0.09)	-0.61 (-1.16; -0.40)	-0.21 (-0.36; 0.04)	*vs. early MS*: <0.01^d^ *vs. PMS*: 0.57^d^ *vs. HC*: <0.01^d^	*vs. 2Y*: 0.6^c^ *vs. early MS*: 0.03^d^ *vs. PMS*: 0.69^d^ *vs. HC*: 0.03^d^
Median PGMVC^*^ (IQR)	-0.76 (-1.07; -0.44)	-0.47 (-0.56; -0.12)	-0.44 (-0.69; -0.20)	-0.57 (-0.97; -0.40)	-0.31 (-0.64; -0.13)	*vs. early MS*: <0.05^d^ *vs. PMS*: 0.68^d^ *vs. HC*: <0.01^d^	*vs. 2Y*: 0.04^c^ *vs. early MS*: 0.7^d^ *vs. PMS*: 0.06 *vs. HC*: 0.8^d^
Median PWMVC^*^ (IQR)	-0.82 (-1.14; -0.55)	-0.49 (-0.86; -0.23)	-0.47 (-0.93; -0.12)	-0.59 (-0.77; -0.39)	-0.29 (-0.59; -0.10)	*vs. early MS*: <0.05^d^ *vs. PMS*: 0.13^d^ *vs. HC*: <0.01^d^	*vs. 2Y*: 0.1^c^ *vs. early MS*: 0.9^d^ *vs. PMS*: 0.08^d^ *vs. HC*: 0.08^d^

2Y-FU: 2-years follow-up; HC: healthy controls; F: female; Gd: gadolinium; IQR: interquartile range; LT-FU: long-term follow-up; M: male; MS: multiple sclerosis; PBVC: percentage brain volume change; PGMVC: percentage gray matter volume change; PWMVC: percentage white matter volume change; T2LV: T2-hyperintense lesion volume

^a^Independent-Samples Kruskal-Wallis test with post-hoc comparisons

^b^Fisher’s exact test

^c^Wilcoxon Signed Rank Test for paired samples

^d^ANCOVA models corrected for age, sex, normalized brain tissue volumes, EDSS and MR scanner (as appropriate)

^§^Moderate Efficacy DMT=azathioprine, interferon beta 1a, or teriflunomide; High Efficacy DMT= fingolimod, natalizumab, ocrelizumab

^#^vs. previous MRI scan

*Variation vs. previous timepoint, results are divided for follow-up duration to obtain annualized values

**Fig. 3:**
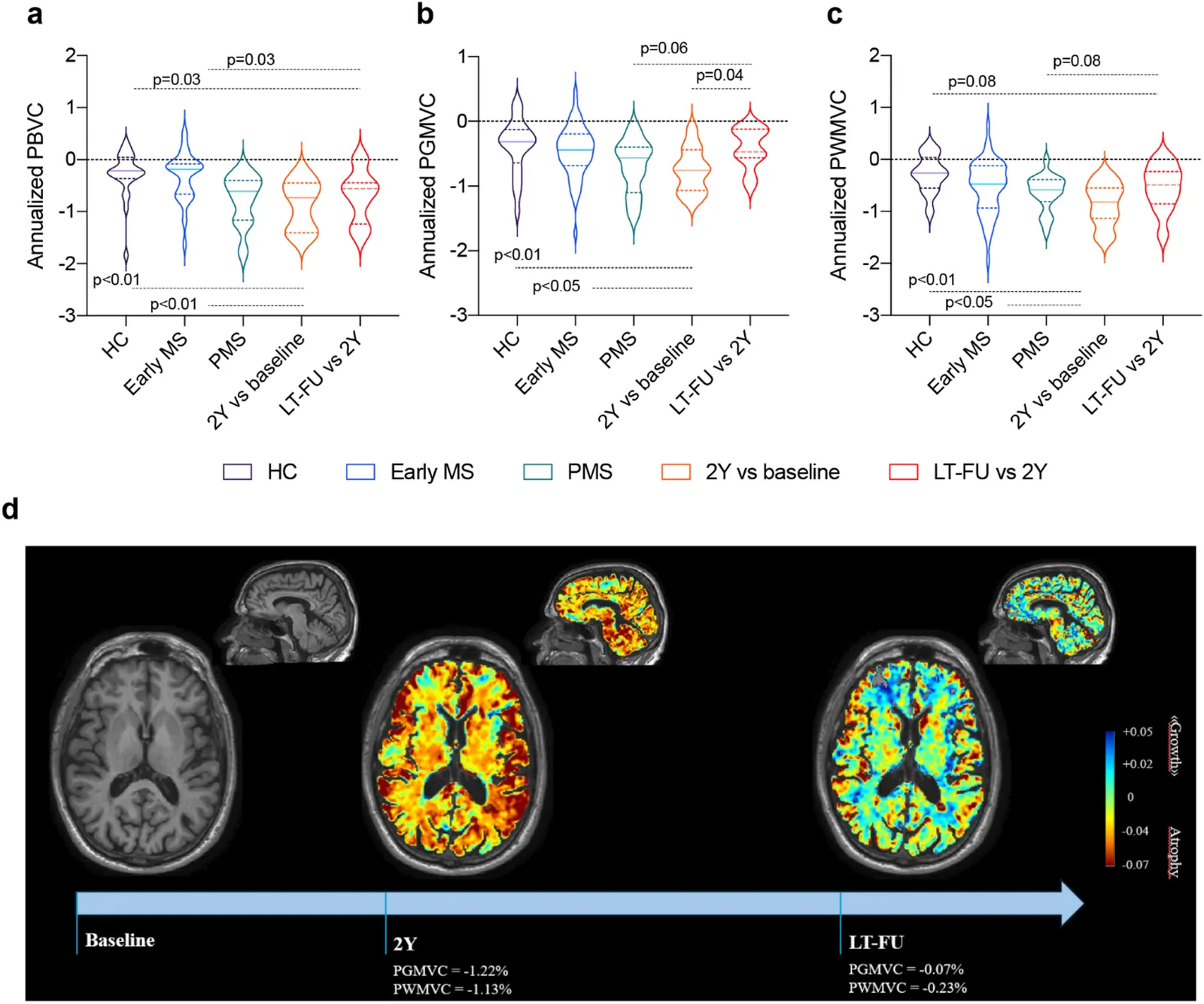
Brain atrophy in STEMS patients and reference cohorts. Violin plots of the annualized percentage brain (**a**), gray matter (**b**) and white matter volume changes (**c**) of the reference cohorts (HC in purple, early MS in blue, and PMS in green) and STEMS-patients at different timepoints (estimated between 2-year (2Y) and baseline visit in orange, and between LT and 2-year follow-up in red). **d** An example of T1-weighted MRI for a patient who received a low-dose of hfNPC with superimposed a colour coded map of the Jacobian determinant in the GM and WM tissues, both at 2-years and LT after transplantation. The integration of the Jacobian determinant over the GM and WM voxels (as extensively described in the Methods section) gave a global estimation of the percentage volume changes. LT-FU/LT: long-term follow-up; 2Y: 2-years follow-up; HC: healthy controls; MS: multiple sclerosis; PMS: progressive MS; PBVC: percentage brain volume change; PGMVC: percentage gray matter volume change; PWMVC: percentage white matter volume change

**Fig. 4:**
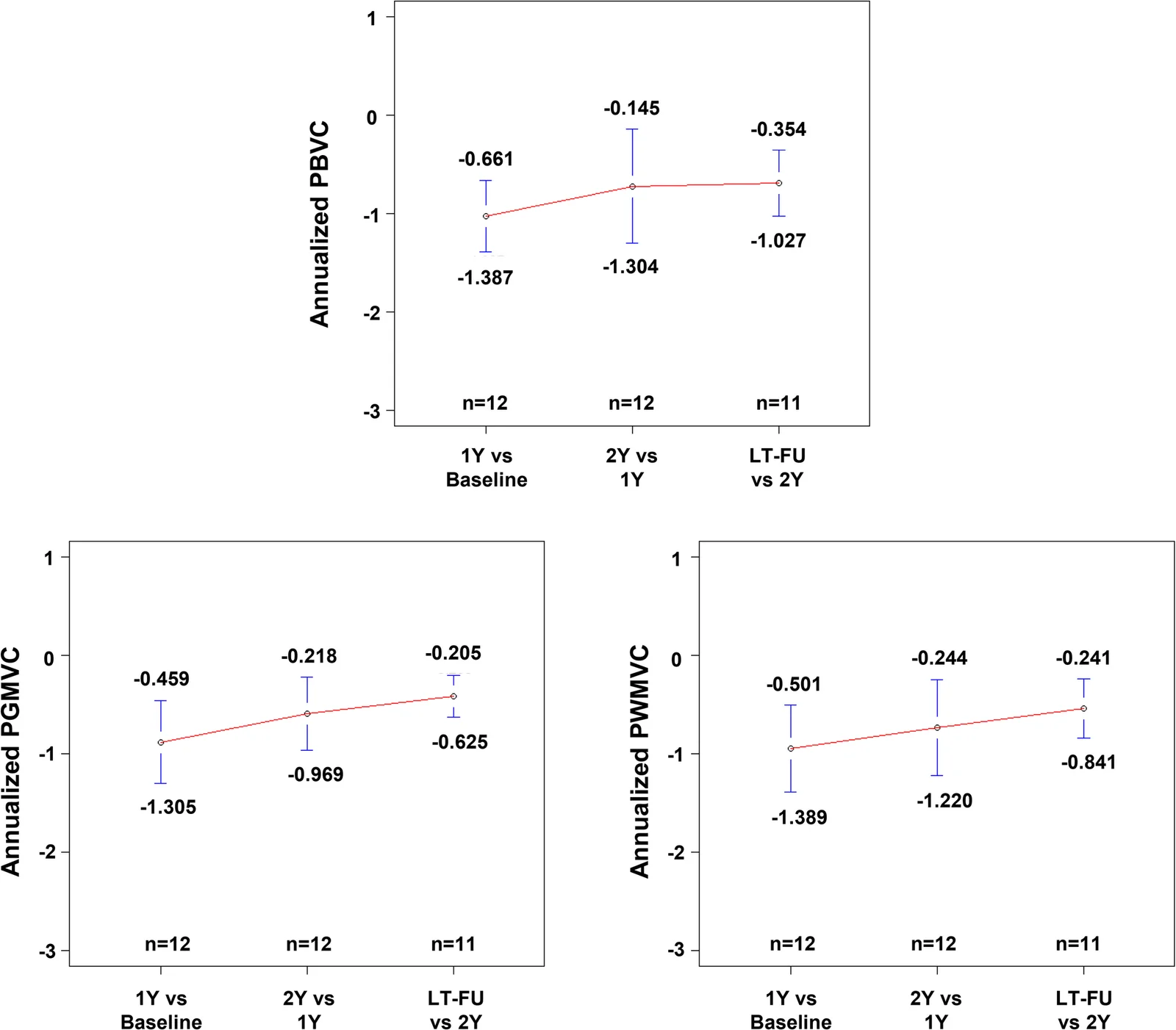
Brain atrophy in STEMS patients after hfNPC transplantation. Median (in red) annualized percentage brain, gray matter and white matter volume changes over time after hfNPC transplantation for STEMS-patients, with 95% confidence interval (in blue). 1Y: 1-year follow-up; 2Y: 2-years follow-up; LT: long-term follow-up; PBVC: percentage brain volume change; PGMVC: percentage gray matter volume change; PWMVC: percentage white matter volume change

No patient showed Gd-enhancing lesions during the LT-FU. Between 2Y-FU and LT-FU, STEMS-patients showed a statistically significant reduction in the annualized rate of gray matter atrophy (PGMVC, *P* = 0.04, Fig. 3b), while no statistically significant differences were found for WM atrophy (PWMVC, *P* = 0.1, Fig. 3c) and brain atrophy (PBVC, *P* = 0.6, Fig. 3a). A patient-level representation of PBVC, PGMVC, and PWMVC at 2Y-FU and LT-FU are reported in Additional file 1. After hfNPC transplantation, STEMS patients exhibited a trend toward lower annualized rates of brain (*P* = 0.6), GM (*P* = 0.04), and WM atrophy (*P* = 0.1) during the post-transplantation follow-up period (Figs. 3d and 4). Considering that 4 out 11 patients started a DMT during the LT-FU, we performed a descriptive sub-analysis evaluating the differences between PBVC, PGMVC and PWMVC between treated and untreated STEMS patients, to explore whether the lower rate of atrophy observed in the LT-FU could be due to the introduction of DMTs. Our analysis showed no significant difference of PBVC between treated and untreated patients. On the contrary, patients who did not receive DMTs in the LT-FU seem to have a significant lower rate of GM and WM atrophy compared to those who underwent therapy (LT-FU median [IQR] PGMVC in treated − 0.55 [-0.88; -0.49] vs. untreated patients − 0.19 [-0.47; -0.09], *p* = 0.04. LT-FU median [IQR] PWMVC in treated − 0.93 [-1.34; -0.71] vs. untreated patients − 0.26 [-0.49; -0.01], *p* < 0.01).

No significant correlation was found between annualized PBVC (adjusted R^2^ = 0.33, *P* = 0.3), PGMVC (adjusted R^2^ = 0.09, *P* = 0.8) and PWMVC (adjusted R^2^ = 0.24, *P* = 0.5) at LT-FU and number of injected hfNPCs.

Since trial design did not include a standardized longitudinal MRI assessment prior to hfNPC transplantation to estimate individual pre-transplant atrophy trajectories, nor a control group, causal relationship between changes in atrophy rates and the procedure cannot be established. To better contextualize long-term atrophy rates observed after transplantation in our patients, we compared them with those of longitudinal reference cohorts of early MS, PMS and HC. Demographic and MRI findings of STEMS-patients and reference cohorts (early MS, PMS and HC) are reported in Table 3. At 2Y-FU, STEMS-patients showed significantly higher annualized atrophy rates in whole brain (*P* < 0.01 vs. early MS, *P* < 0.01 vs. HC), GM (*P* < 0.05 vs. early MS, *P* < 0.01 vs. HC) and WM (*P* < 0.05 vs. early MS, *P* < 0.01 vs. HC) compared to the early MS and HC reference cohorts (indicated by lower PBVC, PGMVC and PWMVC distributions, Fig. 3a-c). However, at the LT-FU, they reached annualized GM and WM atrophy rates that were not significantly different from those observed in early MS patients (*P* = 0.7 for GM, *P* = 0.9 for WM) and age-matched HC (*P* = 0.8 for GM, *P* = 0.08 for WM, Fig. 3a-c). When comparing atrophy rates between STEMS-patients and a reference cohort of PMS patients - who had comparable age and disease duration but lower disability as assessed by EDSS (Table 3) - no significant differences were found at 2Y-FU (*P* = 0.7 for GM, *P* = 0.1 for WM). Interestingly, at LT-FU there was a trend toward a lower GM and WM atrophy rates in STEMS patients compared to PMS (*P* = 0.06 for GM, *P* = 0.08 for WM). No differences in whole brain volume were observed between STEMS-patients and PMS at either timepoints (*P* = 0.6 for 2Y-FU, *P* = 0.7 for LT-FU. Figure 3a-c). The inclusion of phenotype in the sensitivity analysis did not materially modify the results, and the level of statistical significance remained unchanged (GM at LT-FU: *P* = 0.07; WM at LT-FU: *P* = 0.09; whole brain volume: *P* = 0.8 and *P* = 0.9 at 2Y-FU and LT-FU, respectively).

### Plasma biomarkers

Plasma biomarkers of neurodegeneration and inflammation were evaluated longitudinally in 8 out of 12 patients. Serum levels of GFAP decreased at LT-FU (mean ± SD: 151 ± 64.5 pg/ml) compared to baseline (mean ± SD: 199.9 ± 89 pg/ml, *P* = 0.05) and 2-year FU (mean ± SD: 253.2± 131.2 pg/ml; *P* = 0.01). For NfL, tau and UCH-L1, no statistically significant differences were observed between LT-FU (mean ± SD Nfl: 144.3 ± 357.4 pg/ml; tau: 5.5 ± 1 pg/ml; mean ± SD UCH-L1: 30.6 ± 28.9 pg/ml) and baseline (mean ± SD NfL 18.9 ± 9.8 pg/ml, *P* = 0.64; tau: 13.6 ± 15.5 pg/ml, *P* = 0.46; mean ± SD UCH-L1: 61.1 ± 84.6 pg/ml, *P* = 0.11), nor between LT-FU and 2Y-FU (mean ± SD NfL: 24.4 ± 12.2 pg/ml, *P* = 0.84; tau: 25.2 ± 49.4 pg/ml, *P* = 0.99; mean ± SD UCH-L1: 54.17 ± 55.33 pg/ml, *P* = 0.11).

## Discussion

The LT-FU data presented in this study provide valuable insights into the safety profile of hfNPC transplantation and support the hypothesis of potential neuroprotective effects in patients with PMS, although these findings will require confirmation in larger, adequately powered studies. Despite the limitations related to the retrospective design and sample size, our results represent a significant, albeit preliminary, step forward in validating the therapeutic potential of hfNPCs, which had previously shown promise in preclinical models and initial phase I clinical trials [8, 9]. The first and very reassuring result is that we did not record any AEs related to the transplantation procedure over a median follow-up of 5.7 years. As detailed in Table 2, one patient developed a monoclonal gammopathy of undetermined significance IgG kappa at low risk of progression. This AE was considered unlikely related to the procedure since it is typically detected as an incidental finding in 3% of the general population over the age of 50 years [20]. Another patient experienced grade 1 hypertransaminasemia during teriflunomide treatment. The known hepatic risk, as indicated in the EU risk management plan for teriflunomide, makes it unlikely related to the STEMS study procedure. One patient developed ductal carcinoma in situ of the breast, the most commonly diagnosed cancer worldwide [21]. This AE was considered unlikely related to hfNPCs based on epidemiological data, the concurrent immunosuppressive treatment and the localization of the tumor outside the CNS. This adult patient has a positive familiar history for breast cancer and received three doses of ocrelizumab, a monoclonal antibody associated with an increased risk of malignancies, including breast cancer, as indicated in the EU risk management plan. In addition, a diagnostic lumbar puncture performed three months post-transplantation for safety monitoring did not demonstrate the persistence of hfNPCs previously injected into the patient’s CSF [8]. Finally, one patient showed an increase in the size of a previously known hepatic angiomyolipoma. Since the angiomyolipoma was already present before the hfNPC transplantation and preclinical in vivo safety study did not reveal a systemic dissemination of intrathecally administrated hfNPCs [8], this AE was considered unlikely related to the procedure. We believe it is important to emphasize that retrospective collection of adverse events is subject to significant recall bias. While major adverse events can be easily identified even through retrospective data collection, the same does not apply to those of lesser severity and relevance. Additionally, as patients were followed as part of routine clinical care, follow-up visits did not occur at uniform intervals, which may have further affected the accuracy and completeness of adverse event documentation.

The second interesting finding in LT-FU analysis was the difference observed between the progression of physical disabilities and cognitive impairment in STEMS patients. Physical disability progressed in LT-FU patients, with a statistically significant worsening of EDSS scores compared to baseline. A result also supported by the worsening of 9-HPT scores, a more sensitive measure for assessing disability progression in non-ambulatory MS patients. While the rate of EDSS change exhibited a trend toward slowing during the first two years post-transplantation compared to the pre-treatment period, this positive effect was not maintained in the LT-FU period. It is essential to interpret these findings cautiously, as clinical worsening in the year prior to transplantation was an inclusion criterion for the phase I study. The non-linear nature of EDSS, particularly in patients with high disability levels, should be also taken into consideration as a possible explanation of our results. In contrast, SDMT score - the most widely recommended neuropsychological test in MS because of its sensitivity, reliability, and predictive validity [22] - remained stable in the LT-FU compared to baseline. The SDMT improvement we previously observed 2-year after transplantation [8] could be attributable to a practice effect during the trial procedures, which vanished in an observational LT study without frequent evaluations.

Despite the absence of significant differences among NfL, tau and UCHL1 longitudinal changes, the observed reduction in GFAP levels may support the hypothesis of a neuroprotective effect mediated by hfNPCs, as it represents a marker of astrocyte activation and injury. Plasma GFAP levels have been shown to correlate with poor clinical outcomes and chronic inflammation in multiple sclerosis and radiologically isolated syndrome [23].

GM plays a pivotal role in cognition as it houses the majority of neuronal cell bodies, synapses, and networks involved in cognitive processing speed, attention, memory and decision making. Consequently, the progressive atrophy of GM is strongly linked to cognitive decline [24]. Cognitive impairment affects 34% to 65% of adults with MS [25, 26], making it one of the most common and clinically relevant symptoms of the disease [22]. MRI data from this study revealed at LT-FU a statistically significant reduction of GM atrophy rate compared to 2 years after transplantation. Emphasizing the importance of these findings, long-term GM and WM atrophy in STEMS patients attained rates that are comparable to those observed in early MS patients and age-matched HC. Despite no statistical differences were detected compared to a reference population of PMS, we observed a nearly significant reduction of long-term GM and WM atrophy in our patients. However, comparisons with external reference cohorts should be interpreted with caution, given differences in MRI acquisition protocols, population characteristics, disability burden, and sample size. In particular, the limited number of subjects in the reference cohorts, particularly in the PMS group, reflects the effort to closely match the populations under analysis, but limits the robustness of between-group comparisons. Importantly, in the absence of a proper control group and a standardized longitudinal MRI assessment before hfNPC transplantation, it is not possible to establish causal relationships between changes in atrophy rates and the procedure. The observed reduction in atrophy rates may therefore reflect, at least in part, the natural evolution of tissue loss at advanced disease stages. Accordingly, these findings should be considered descriptive, exploratory, and hypothesis-generating only. Nevertheless, the observation of a trend toward a lower rate of GM and WM atrophy compared to a PMS population matched for age and disease duration, but with lower disability - thus likely representing a less aggressive disease form than that of STEMS patients – may represent an encouraging preliminary signal that warrants further investigation in adequately powered controlled studies, including the ongoing phase II study. In this context, the relevance of GM atrophy in distinguishing disease phenotypes and predicting disability and cognitive impairment [27] was demonstrated, with reports of a 14 times greater relative increase of GM atrophy in patients with SPMS compared to controls [28]. Similarly, the stabilization of cognitive performance over time, along with the significant reduction in GM volume loss, and the proteomic findings from the phase I study indicating upregulation of pathways involved in axonal development and protection, prompt us to hypothesize a biological activity of transplanted NPCs. A conceivable mechanistic explanation might rely on the physiological role of human NPCs in the adult brain. As a matter of fact, such cells are located throughout life within the subventricular zone [29–31], a region of the brain typically affected in MS patients due to the presence of demyelinating lesions. Among periventricular NPCs, some are able to secrete trophic factors that are essential for a proper functioning of the striatal circuits considered essential in supporting cognitive functions [32]. Based on our exploratory results it can be hypothesized that hfNPCs transplantation may prevent cognitive decline in MS through a neuroprotective effect on GM, however, this assumption requires confirmation through controlled comparative studies. From a mechanistic point of view, we can speculate that the presence of transplanted NPCs may contribute over time to modify the microenvironment via the release of soluble molecules that, in turn, promote sustained endogenous repair mechanisms driven by CNS-resident cells surviving to the inflammatory demyelinating insult. However, the study design, the small sample size, and the absence of a proper control population do not allow for conclusions to be drawn in this regard, making the interpretation of efficacy data purely speculative. The observation of an increased T2-hyperintense brain lesion volume in some of the patients at LT-FU remains a relevant a concern. This result might suggest that while cell transplantation may provide neuroprotection via the release of neuroprotective molecules, it might not control and could potentially influence inflammatory processes. Despite the correlation analyses performed in the phase I study did not demonstrate a clear association between the number of transplanted hfNPCs and the occurrence or volume of new T2 lesions, the limited sample size and consequent low statistical power preclude definitive conclusions. Therefore, a potential effect related to the transplantation procedure, particularly at higher cell doses, cannot be excluded. In light of these considerations, and adopting a precautionary approach, the total cell dose selected for the phase II trial has been divided into two separate administrations. This dosing strategy is intended to mitigate potential inflammatory responses and to enable close clinical, radiological, and laboratory monitoring between administrations, allowing for early detection and management of any emerging safety signals. Furthermore, administering the treatment in two sequential doses may help sustain over time the potential neuroprotective and pro-regenerative effects of the transplanted cells, thereby maximizing their therapeutic impact.

This study has several limitations that should be carefully considered. First, it is a retrospective observational study based on data collected during routine clinical practice, without standardized timing of assessments, and conducted on a small patient population. Second, the comparisons with external reference cohorts should be interpreted with substantial caution. Although reference populations were selected to provide the closest possible match, they were not fully comparable to the STEMS cohort. Differences in study design, MRI acquisition protocols, and disability burden may all have influenced the observed findings and limit the robustness of between-group comparisons. In particular, EDSS scores of the control PMS group were lower than those of the STEMS patients, reflecting the challenge of identifying external cohorts with similarly advanced disability levels and long-term follow-up data in observational studies. Moreover, the relatively small number of subjects included in the external reference cohorts, especially in the PMS population, further limits statistical power and the generalizability of these comparisons.

Importantly, the absence of a proper internal control group and the lack of standardized longitudinal MRI assessments prior to hfNPC transplantation preclude reliable estimation of individual pre-treatment atrophy trajectories and prevent any causal interpretation of the observed MRI changes. Moreover, a proportion of patients in both the STEMS and PMS control group received DMTs during follow-up, with a similar treatment rate between the two groups. A sub-analysis comparing treated and untreated STEMS patients did not reveal any significant differences. Nonetheless, this parameter should be taken into account, as it may have influenced MRI measures. The relatively small sample size limited our ability to perform reliable subgroup analyses based on additional clinical variables, such as pre-treatment disease activity. Disease phenotype (PPMS vs. SPMS) was not analyzed through stratified subgroup analyses but was included as a covariate in the model. Separate subgroup analyses would likely have led to insufficient statistical power and unstable estimates. Future studies involving larger, well-characterized cohorts are warranted to more robustly assess the contribution of these factors to atrophy progression. Given the exploratory nature of the analyses and the limited sample size, no adjustments for multiple comparisons were applied; therefore, the results should be interpreted with appropriate caution. Finally, although different MRI scanner field strengths were used for the reference population, the analyses accounted for scanner variability to minimize potential biases. Moreover, the longitudinal design, which allowed for within-subject comparisons over time with a key focus on maintaining consistency between baseline and follow-up scans within each individual, supported meaningful comparisons across different populations further mitigating potential scanner-related biases.

## Conclusions

In conclusion, although hfNPC transplantation appears safe in the medium term, its efficacy remains to be consistently demonstrated. Preliminary data supporting a potential for reducing neurodegeneration in PMS patients warrant further investigation. The central question now shifts to whether these therapies can produce measurable functional benefits and slow disease progression. Progress in the field is cautious but steady: ongoing and future studies with larger patient cohorts, and randomized control groups will be essential to provide definitive answers. As recently approved by the Italian regulatory authority (EUCT number 2024-511028-15-00), we are conducting a phase II, randomized, patient and assessor blind, multicenter study comparing hfNPC transplantation to sham procedure in PMS that will provide additional and stronger results about the efficacy of this therapeutic approach. Should these efforts prove successful, NPC transplantation could emerge as a valuable addition to the therapeutic arsenal for neurodegenerative and demyelinating diseases such as MS, potentially in combination with other treatments to address both the neurodegenerative and immune-mediated components of the disease.

## Supplementary Information

Below is the link to the electronic supplementary material.

Supplementary Material 1.

## Data Availability

Pseudonymized participant data supporting the findings reported in this article can be shared in compliance with current data protection regulations by the European Union. Data sharing requires a current and positive vote by the requestor’s competent ethics committee. All proposals should be directed to the corresponding author.

## Abbreviations

AE: Adverse event
AZA: Azathioprine
CNS: Central nervous system
CSF: Cerebrospinal fluid
CTX: Cyclophosphamide
DH: Dominant hand
DMT: Disease modifying therapy
DMF: Dimethyl Fumarate
EDSS: Expanded Disability Status Scale
ETL: Echo train length
FA: Flip angle
FLAIR: Fluid-attenuated inversion recovery
FOV: Field of view
FTY: Fingolimod
FU: Follow-up
GM: Gray matter
HC: Healthy controls
hfNPCs: Human fetal NPCs
IQR: Interquartile Range
IR–FSPGR: Inversion recovery fast spoiled gradient echo
LT: Long-term
MPRAGE: Magnetization-prepared rapid gradient echo
MRI: Magnetic resonance imaging
MS: Multiple sclerosis
MTX: Methotrexate
NfL: Neurofilament light chain
NPCs: Neural precursor cells
NSA: Number of signal averages
OCR: Ocrelizumab
OCT: Optical coherence tomography
ON: Optic neuritis
PBVC: Percentage brain volume change
PGMVC: Percentage gray matter volume changes
PMS: Progressive multiple sclerosis
PWMVC: Percentage white matter volume changes
pRNFL: Peripapillary retinal nerve fiber layer
RGP: Resorufin ß-d-galactopyranoside
RTX: Rituximab
SBG: Streptavidin-ß-galactosidase
SDMT: Symbol Digit Modalities Test
T2LV: T2-hyperintense lesion volume
TC: Treatment cohort
TE: Echo time
TFE: Turbo field echo
TI: Inversion time
TR: Repetition time
TSE: Turbo spin echo
UCH-L1: Ubiquitin C-terminal hydrolase-L1
WM: White matter
